# Dataset of Vietnamese students’ academic perfectionism and school alienation

**DOI:** 10.1016/j.dib.2021.107463

**Published:** 2021-10-09

**Authors:** Thanh-Thao Thi Phan, Linh-Chi Nguyen, Ngoc-Quang Nguyen, Yen-Chi Nguyen

**Affiliations:** a Thanh Do University, Hanoi 100000, Viet Nam; b EdLab Asia Educational Research and Development Centre, Hanoi 100000, Viet Nam; c Utrecht University, Utrecht 3584 CS, the Netherlands

**Keywords:** Student academic perfectionism, Meritocracy belief, Classroom competitiveness, Vietnam, School alienation, Parental conditional regard, Teacher conditional regard, Contingent self-worth

## Abstract

Across the steadily superseding world, the younger generation is coming under pressure for an increase in the standard and a highly growing demand on their life themselves. This could lead to a variety of problems, including academic perfectionism and school alienation. To gain more insights into these phenomena, we conducted two research projects on students from eight upper secondary schools in Ho Chi Minh City, Vietnam using online surveys, and obtained two datasets. Dataset A covers (i) the level of students' perfectionism; (ii) belief in school meritocracy; (iii) The competitiveness among students; and (iv) the intrinsic motivation to achieve. Dataset B contains students' self-reports about (i) their perceptions of parents' and teachers' academic conditional regard; (ii) academic contingent self-worth; and (iii) school alienation. The numbers of respondents of dataset A and dataset B are 2942 and 2970, respectively.

**Specifications Table**

**Table:** 

Subject	Education, Psychology
Specific subject area	Education Management; Educational Psychology; Developmental Psychology
Type of data	Raw data in excel file, and analyzed data in tables
How data were acquired	Data was gathered using an online survey generated in Google Form (a web-based survey platform)
Data format	Raw Analyzed
Parameters for data collection	The paper focuses on students’ psychological issues, using self-report data from upper secondary school students (Grade 10–12) from southeast areas in Vietnam.
Description of data collection	Before the survey was delivered directly to random students, we introduced the purpose as well as the content of our survey to those schools’ teachers. After that, teachers explained the consent form to students and supported students to fulfill the questionnaires.
Data source location	Information is collected from upper secondary student schools in Ho Chi Minh City (Latitude 10°49′22.87"N, Longitude 106°37′46.74"E), Vietnam
Data accessibility	Repository name: Mendeley Data Data identification number: Direct URL to dataset A: https://data.mendeley.com/datasets/jf43t395zs/4, Mendeley Data, V4 Direct URL to dataset B: https://data.mendeley.com/datasets/jsbbfpvf6p/2, Mendeley Data, V2

**Value of the Data**

• Dataset A and B contribute to extending the data of academic perfectionism and to the knowledge of the prevalence and distribution of school alienation across demographic groups among Vietnamese upper secondary students.
• The datasets can help teachers, school administrators, and education researchers formulate comprehensive policies, which partly minimize the negative effect of perfectionism and school alienation.
• Dataset A can help to validate a new instrument to measure academic perfectionism. At the same time, psychometricians could exploit dataset B to construct psychological scales assessing school alienation, parents' and teachers' academic conditional regard, and academic contingent self-worth. With a view from a developing country, gathering with datasets acquired from other cultural contexts, these two datasets may help to expand our understanding of the cross-cultural divergences and convergences in terms of each variable and their associations.
• Dataset A provides an examination of the relationship between academic perfectionism and intrinsic motivation, students’ belief in school meritocracy, and students’ perceptions of class competitiveness.
• Dataset A performs as a starting point to explore students’ belief in school meritocracy, an essential concept regarding inequality justification. A better understanding of this topic will help teachers, school administrators, and policymakers to aware of students’ mindsets on inequality and tackle the problem.
• Researchers could analyze dataset B to investigate the relationships among school alienation, parents' and teachers' academic conditional regard, academic contingent self-worth, and GPA.

## 1 Data Description

Perfectionism is a topic that has received a lot of attention in the psychology field. It was first mentioned as a pathological trait, which obscures the therapy process, but later studied as a trait that can also appear in the non-clinical population. Many researchers also argue about the importance of studying perfectionism under a specific domain of life [1]. On the other hand, school alienation, namely negative attitudes towards schooling, is the most common reason for dropping out of school among Vietnamese children and adolescents [2]. In addition, school alienation can be the culprit for changing everyday learning habit and leads to problematic social behaviors [3]. Researchers suggested that the negative interaction between students and their parents and teachers in the academic domain might be the antecedents of this psychological phenomenon. In academic settings, these two phenomena recently occurred significantly and should be more highlighted, especially in the era of learning sustainability [4]. To ensure the confidentiality of participants, we coded school names using capital letters from A to G (for seven schools in dataset A), and roman numbers (for eight schools in dataset B). The two reported datasets are a part of a project aim to explore the contemporary psychological issues in Vietnamese students.

Regarding dataset A (Students’ perfectionism), the survey comprised five main parts: (i) The students’ demographic information; (ii) The students’ perfectionism level in academic backgrounds; (iii) Students’ belief in school meritocracy; (iv) Students’ perception of classroom competitiveness and (v) Students’ intrinsic motivation in terms of achievement. The students’ demographic information was presented in Table 1. Table 2 described students’ frequency of participating in voluntary activities and students’ level of perfectionism. Students' opinions on school meritocracy, competitiveness, and intrinsic motivation were displayed in Table 3.

**Table 1:** Demographic of dataset A.

Variables	Frequency	Percent (%)
***School name***	***2942***	***100***
A	397	13.5
B	434	14.8
C	399	13.6
D	420	14.3
E	485	16.5
F	420	14.3
G	387	13.2

***Gender***	***2942***	***100***
Male	1561	53.1
Female	1381	46.9

***Year of Birth***	***2942***	***100***
2002	1093	37.2
2003	968	32.9
2004	881	29.9

***GPA***	***2942***	***100***
5.1.- 6.0	410	13.9
6.1 - 7.0	1032	35.1
7.1 - 8.0	993	33.8
8.1 - 9.0	507	17.2

**Table 2:** Descriptive statistics of students’ voluntary activities and the level of students’ perfectionism (*N* = 2942).

				Mean			
Variables	Range	Min	Max	Statistic	Std. Error	Std. Deviation	Variance
5.1 Mental Health	4	1	5	2.28	0.024	1.281	1.641
5.2 Physical Health	4	1	5	3.45	0.023	1.268	1.608
5.3 Environmental	4	1	5	3.11	0.022	1.207	1.457
5.4 Education	4	1	5	2.57	0.023	1.226	1.502
5.5 Gender Equity	4	1	5	2.38	0.022	1.199	1.438
5.6 Poverty Eradication	4	1	5	3.07	0.023	1.246	1.553
5.7 Peace and Justice	4	1	5	3.45	0.023	1.225	1.500
6.1 If I fail at school, I am a failure as a person	4	1	5	3.36	0.019	1.050	1.102
6.2 If someone does a task at school better than me, then I feel like I failed at the whole task	4	1	5	3.42	0.020	1.110	1.233
6.3 My parents do not always expect me to be perfect in everything I do at school	4	1	5	2.11	0.022	1.181	1.394
6.4 If I do not do well all the time at school, people will not respect me	4	1	5	3.50	0.019	1.021	1.043
6.5 There are people in my life who expect me to be a perfect student	4	1	5	3.49	0.023	1.241	1.541
6.6 The fewer mistakes I make at schoolwork, the more people will like me	4	1	5	3.40	0.019	1.008	1.015
6.7 I set higher goals for myself at school than most people	4	1	5	3.41	0.019	1.012	1.024
6.8 My family expects me to be a perfect student	4	1	5	3.37	0.021	1.160	1.346
6.9 I have extremely high goals for my learning	4	1	5	2.95	0.026	1.422	2.021
6.10 People expect more from my learning ability than I am able to give	4	1	5	3.49	0.019	1.021	1.043
6.11 Other students seem to accept lower standards from themselves than I do	4	1	5	3.56	0.020	1.074	1.153
6.12 Other people think that I have failed if I do not do my very best at school all the time	4	1	5	3.57	0.018	0.995	0.989
6.13 Other people always expect me to be perfect	4	1	5	3.39	0.019	1.045	1.092
6.14 I expect higher performance in my daily learning tasks than most people	4	1	5	3.44	0.020	1.083	1.173
6.15 People around me expect me to be great at everything at school	4	1	5	3.20	0.019	1.017	1.035
6.16 My teachers expect my work to be perfect	4	1	5	3.19	0.020	1.081	1.169
6.17 I am always expected to do my learning tasks better than others	4	1	5	3.33	0.020	1.082	1.170
6.18 I feel that people ask too much of me	4	1	5	3.43	0.020	1.085	1.178

**Table 3:** Descriptive statistics of students' opinions on school meritocracy, classroom competitiveness and students’ intrinsic motivation (*N* = 2942).

				Mean			
Variables	Range	Min	Max	Statistic	Std. Error	Std. Deviation	Variance
7.1 Everyone has the same chances to succeed at school	4	1	5	3.46	0.018	0.962	0.925
7.2 Efforts and hard-working hard is not enough, there are other factors affecting school achievements	4	1	5	2.58	0.023	1.267	1.606
7.3 At school, students who obtain poor grades are those who have not worked enough	4	1	5	3.41	0.019	1.009	1.018
7.4 At school, students are rewarded (they obtain good grades, praise) for their efforts	4	1	5	3.53	0.019	1.013	1.027
7.5 At school, children obtain the grades they deserve	4	1	5	3.65	0.018	0.975	0.952
7.6 At school, students who obtain good grades are those who have worked hard	4	1	5	3.74	0.020	1.068	1.140
8.1 Most students want their work to be better than their friends' work	4	1	5	3.77	0.017	0.946	0.894
8.2 Students compete to see who can do the best work	4	1	5	3.89	0.020	1.069	1.144
8.3 A few of the class members always try to do better than the others	4	1	5	4.03	0.016	0.873	0.762
8.4 Students seldom compete with one another	4	1	5	1.91	0.020	1.078	1.163
8.5 Students feel left out unless they compete with their classmates	4	1	5	3.71	0.016	0.857	0.735
9.1 Because I experience pleasure and satisfaction while learning new things	4	1	5	3.43	0.021	1.141	1.301
9.2 For the pleasure I experience while surpassing myself in my studies	4	1	5	3.54	0.020	1.079	1.164
9.3 For the pleasure I experience when I discover new things never seen before	4	1	5	3.22	0.021	1.166	1.359
9.4 For the pleasure that I experience while I am surpassing myself in one of my personal accomplishments	4	1	5	3.63	0.018	0.972	0.944
9.5 For the pleasure that I experience in broadening my knowledge about subjects which appeal to me	4	1	5	3.76	0.019	1.040	1.082
9.6 For the satisfaction I feel when I am in the process of accomplishing difficult academic activities	4	1	5	3.28	0.023	1.254	1.573
9.7 Because my studies allow me to continue to learn about many things that interest me	4	1	5	3.72	0.018	0.977	0.954
9.8 Because high school allows me to experience a personal satisfaction in my quest for excellence in my studies	4	1	5	3.56	0.017	0.909	0.827

Since the scales used are self-administered, we carried out a principal axis factoring with oblique rotation method to test whether the items reflected a shared structure. One factor was extracted for all of the scales. For the Academic Perfectionism scale, the factor loadings of items ranged from  0.566 to 0.844, with the cumulative explained variance was 50.008%. The Cronbach's alphas of sub-scales were between 0.77 and 0.913, and the whole scale was 0.936.

The Belief in Meritocracy scale had the factor loadings of items ranged from 0.52 to 0.826, with the cumulative explained variance was 48.911%. After deleting the item Student_Try, which had factor loading lower than 0.3, the Perceived Classroom Competitiveness scale had four items with factor loadings varied from 0.571 to 0.655, and the cumulative explained variance was 38.235%. The factor loadings of the Intrinsic Learning Motivation scale's items varied from 0.519 to 0.749 and the cumulative explained variance was 38.235%, 40.910%. The Cronbach's alpha of each scale is 0.839, 0.708, and 0.837, respectively.

Regarding dataset B (Students’ school alienation), a similar approach has been adopted with eight upper secondary schools in Ho Chi Minh City. This dataset consists of five main parts. The first one is the participants' demographic characteristics (Table 4).

**Table 4:** Demographic characteristics of dataset B.

Variables	Frequency	Percent (%)
***Gender***	***2970***	***100***
Male	1438	48.40
Female	1491	50.20
Prefer not to reveal	41	1.40

***School***	***2970***	***100***
I	535	18.00
II	211	7.10
III	264	8.90
IV	133	4.50
V	561	18.90
VI	194	6.50
VII	538	21.50
VIII	434	14.60

***Grade***	***2970***	***100***
Grade 10	927	31.20
Grade 11	899	30.30
Grade 12	1144	38.50

***GPA***	***2970***	***100***
Under 3.5	0	0.00
From 3.5 to under 5.0	0	0.00
From 5.0 to under 6.5	77	2.60
From 6.5 to under 8.0	1791	60.30
Above 8.0	1102	37.10

***Mother (or stepmother) 's education level***	***2970***	***100***
Primary school	0	0.00
Secondary school	0	0.00
High school	1049	35.30
Graduate	1337	45.00
Postgraduate	584	19.70

***Mother (or stepmother) 's occupation***	***2970***	***100***
Managers	178	6.00
Professionals	287	9.70
Technicians and associate professionals	306	10.30
Clerical support workers	808	27.20
Service and sales workers	538	18.10
Skilled agricultural, forestry and fishery workers	225	7.60
Craft and related trades workers	473	15.90
Plant and machine operators, and assemblers	0	0.00
Elementary occupations	0	0.00
Armed forces occupations	155	5.20

***Mother (or stepmother) 's monthly average income***	***2970***	***100***
Under 5 million VND	0	0.00
From 5 to under 10 million VND	155	5.20
From 10 to under 15 million VND	1370	46.10
From 15 to under 20 million VND	1308	44.00
Above 20 million VND	137	4.60

***Father (or stepfather) 's education level***	***2970***	***100***
Primary school	0	0.00
Secondary school	25	0.80
High school	820	27.60
Graduate	1728	58.20
Postgraduate	397	13.40

***Father (or stepfather) 's occupation***	***2970***	***100***
Managers	204	6.90
Professionals	217	7.30
Technicians and associate professionals	572	19.30
Clerical support workers	607	20.40
Service and sales workers	0	0.00
Skilled agricultural, forestry and fishery workers	425	14.30
Craft and related trades workers	627	21.10
Plant and machine operators, and assemblers	40	1.30
Elementary occupations	0	0.00
Armed forces occupations	278	9.40

***Father (or stepfather) 's monthly average income***	***2970***	***100***
Under 5 million VND	0	0.00
From 5 to under 10 million VND	316	10.60
From 10 to under 15 million VND	560	18.90
From 15 to under 20 million VND	1645	55.40
Above 20 million VND	449	15.10
*Note: N* = 2970.		

The second part (Table 5) refers to students' perception of their parental academic conditional regard (, including parental academic conditional positive regard (PCPR) and parental academic conditional negative regard (PCNR). PCPR is a parenting practice in which a parent expresses more affection and attention towards his or her children when they meet the parent's academic expectations [5]. Meanwhile, a parent practicing PCNR withdraws their appreciation and care for his or her children when they fail in achieving an excellent academic result [5].

**Table 5:** Descriptive statistics of students' reports on parents’ academic conditional regard (*N* = 2970).

				Mean			
Variables	Range	Minimum	Maximum	Statistic	Std. Error	Std. Deviation	Variance
14.1.							
I feel that when (or if) I study hard, my parents will appreciate me more	2	3	5	4.11	.014	.754	.569
14.2.							
I feel that when (or if) I do well in my exam, my parents will accept me more	2	3	5	4.01	.015	.798	.637
14.3.							
I feel that when (or if) I have a good academic achievement, my parents will care more or pay more attention to me	2	3	5	3.91	.014	.784	.614
14.4.							
I feel that when (or if) I study well, my parents will love me more	4	1	5	3.92	.016	.885	.784
14.5.							
I feel that when (or if) I have a good academic achievement, my parents will be more gentle and warm with me.	4	1	5	3.89	.018	.977	.956
15.1.							
I feel that when (or if) I do not study hard, my parents will appreciate me less	4	1	5	3.97	.017	.948	.899
15.2.							
I feel that when (or if) I do not do well in my exam, my parents will accept me less	4	1	5	3.99	.017	.931	.866
15.3.							
I feel that when (or if) I do not have a good academic achievement, my parents will care less or pay less attention to me.	4	1	5	4.02	.016	.866	.751
15.4.							
I feel that when (or if) I do not study well, my parents will love me less	4	1	5	4.02	.018	.957	.917
15.5.							
I feel that when (or if) I do not have a good academic achievement, my parents will be less gentle and warm with me	4	1	5	4.00	.018	.981	.963

The third part of the dataset (Table 6) gives information about student's perception of their teacher's academic conditional regard, including teachers' academic conditional positive regard (TCPR) and teachers' academic conditional negative regard (TCNR) [6]. In the same way with parental academic conditional regard, these two concepts respectively describe situations in which a teacher's provision and withdrawal of his or her respect and attention towards students contingently on what they accomplish at school.

**Table 6:** Descriptive statistics of students' reports on teachers’ academic conditional regard (*N* = 2970).

				Mean			
Variables	Range	Minimum	Maximum	Statistic	Std. Error	Std. Deviation	Variance
16.1. I feel that when (or if) I study hard, my teachers will appreciate me more	4	1	5	4.05	.016	.850	.723
16.2. I feel that when (or if) I do well in my exam, my teachers will accept me more	4	1	5	4.08	.017	.915	.837
16.3. I feel that when (or if) I have a good academic achievement, my teachers will care more or pay more attention to me	4	1	5	4.18	.016	.849	.721
16.4. I feel that when (or if) I study well, my teachers will like me more	4	1	5	4.21	.016	.899	.807
16.5. I feel that when (or if) I have a good academic achievement, my teachers will be more friendly with me	4	1	5	4.32	.016	.852	.726
17.1. I feel that when (or if) I do not study hard, my teachers will appreciate me less	4	1	5	4.21	.014	.753	.567
17.2. I feel that when (or if) I do not do well in my exam, my teachers will accept me less	2	1	3	1.46	.011	.596	.355
17.3. I feel that when (or if) I do no have a good academic achievement, my teachers will care less or pay less attention to me	4	1	5	4.22	.014	.778	.605
17.4. I feel that when (or if) I do not study well, my teacher will like me less	4	1	5	4.28	.015	.809	.655
17.5. I feel that when (or if) I do no have a good academic achievement, my teachers will be less friendly with me	4	1	5	4.16	.015	.829	.687

Another part of this dataset (Table 7) addresses students' academic contingent self-worth (CSW) which means the degree to which students' positive and negative feelings towards their selves are dependent on their academic performance [7].

**Table 7:** Descriptive statistics of students' reports on self-worth academic conditional regard (*N* = 2970).

				Mean			
Variables	Range	Minimum	Maximum	Statistic	Std. Error	Std. Deviation	Variance
18.1. I feel good about myself when (or if) I have a good academic achievement	4	1	5	4.12	.016	.896	.803
18.2. I feel bad about myself when (or if) I do not have a good academic achievement	3	2	5	4.60	.011	.588	.346
18.3. My feelings toward myself are affected by my academic achievement	4	1	5	4.30	.015	.813	.662
18.4. I feel more valuable when (or if) I have a good academic achievement	3	2	5	4.28	.013	.723	.523
18.5. My sense of self-worth is reduced when my academic results are not good	2	3	5	4.38	.012	.643	.414

The last part of the dataset cover students’ self-reports about their attitude toward school, which implies the student's school alienation (SAL).

The Cronbach'alpha of the School Alienation scale, Parental Conditional Regard scale, Teacher Conditional Regard scale, and the Academic Contingent Self-worth are 0.407, 0.101, 0.171, 0.220, respectively. The same method which was used to test the validity of dataset A's scales was performed. Nine factors were extracted with the School Alienation scale, but only one factor had its eigenvalue above 1. Therefore, the analysis was repeated with the number of factors constrained to one. Only six items had factor loadings higher than 0.3, specifically, ranged from 0.301 to 0.386. The one factor explained 4.388% of the variance.

The Parental Conditional Regard scales ended up with four factors, but only 4 items remained (as the rest had low factor loadings) with factor loadings varied between 0.319 and 0.383. 4 factors were extracted when examining the Teacher Conditional Regard scale; only 2 items had their factor loadings higher than 0.3, which were 0.301 and 0.321 in particular.  For The Academic Contingency Self-worth, two factors were extracted, but only two items had their factor loadings higher than 0.3, which were 0.421 and 0.356. However, for all of the three above scales, there were not any factors that had eigenvalue greater than 1.

## 2 Experimental Design, Materials and Methods

The researchers designed the surveys on Google Forms with questions commensurate with each dimension in the scale (except demographic questions). These questions followed the 5-point Likert scale (1: totally disagree; 2: somewhat disagree; 3: neither agree nor disagree; 4: somewhat agree; 5: totally agree). Because our respondents were upper secondary students, we had to get permission from their headmasters as well as teachers, and then teachers introduced and instructed their students to complete the questionnaire via online platforms such as mobile phones, computers or tablets. Three or four classes from each school were randomly selected to participate. Also, each student can decide not to take part in the survey or withdraw anytime if they do not want to continue. The data collection occurred in two months, from the 28th of February to the 30th of April 2020. For dataset A, we collected 2942 responses from seven high schools with the population size of more than 21,000 students. Similarly, 2970 responses were obtained from eight different high schools with the population size of more than 25,000 students for dataset B.

Regarding students’ perfectionism (Dataset A), we carried out adapting available scales to measure upper secondary student academic perfectionism [8,9], student's belief in school meritocracy [10], student's competitiveness [11] and student's intrinsic motivation in terms of their achievement [12]. Although there are a few attempts on exploring perfectionism in the academic domain of student life, the instruments failed to reach a compromise. That is, this dataset introduced and described the specific domain for measuring student academic perfectionism (PERF_LEV). In the PERF_LEV factor, we examined student's expectation of their own learning as well as their evaluation of others expectation of their academic performance, for example, “If I fail at school, I am a failure as a person” or “Other people think that I have failed if I do not do my very best at school all the time”.

Inspired by the work of Curran & Hill [13], we want to explore the relationship between student academic perfectionism and their meritocracy belief (MERIT), their perceived classroom competitiveness (COMPET) and their intrinsic learning motivation (INTRINS). In the MERIT factor, we examine their opinions on aspects such as student learning chance, grade or reward at school, whether or not these aspects are determined completely by hard work and ability. The COMPET factor measures the degree to which students view their classmates as competitive rivals. The INTRINS factor discovered students’ innate motivation to achieve in their academic performance. Particularly, we present the influence of student's level of perfectionism over the three factors MERIT, COMPET, INTRINS by the following regression:

PERF_LEV∼β0+β1*(MERIT)+β2*(COMPET)+β3*(INTRINS)+u

We continue to explore the relationship between these three factors (MERIT, COMPET, INTRINS) with student's frequency of participating in voluntary activities (FRE_ACT). These voluntary activities include mental, physical health; environment; education; gender equality; poverty reduction as well as peace and justice. The impact of student's frequency of participating in voluntary activities on the three factors (MERIT, COMPET, INTRINS) can be explored through the following regression:

FRE_ACT∼β0+β1*(MERIT)+β2*(COMPET)+β3*(INTRINS)+u

To determine students’ alienation (Dataset B), we adopted the school alienation scale developed by Morinaj et al. [3]. The scale comprised 24 items equally divided into three subscales that were alienation from learning (AFL) (i.e., the feelings that learning is boring, meaningless, or useless), alienation from teachers (AFT) (i.e., feelings that one is not accepted or care about by teachers), and alienation from classmates (AFC) (i.e., feelings that one does not belong to the class or is not accepted by peer at school). Participants expressed their agreement with each item on a five-point Likert scale, ranging from 1 = strongly disagree to 5 = strongly agree. Several items requiring reverse scoring were marked with asterisks in the dataset file. The sum of items from a subscale quantified the feeling of a student towards the corresponding domain. A higher score showed a higher level of alienation a student experienced.

To measure students' perceptions of parental academic conditional regard, we constructed ten items based on the parental conditional regard scale for the academic domain proposed by Roth et al. [5].

The first five items measured parental academic conditional positive regard (e.g., I feel that when or if I study hard, my parents will appreciate me more). The remaining items assessed students' perception of parental academic conditional negative regard (e.g., I feel that when or if I do not have a good academic achievement, my parents will care less or pay less attention to me). Participants were asked to indicate the degree to which they agreed with each item on a five-point Likert scale, ranging from 1 = strongly disagree to 5 = strongly agree. A higher sum of each group of five items reflected a higher degree to which students experienced parental conditional positive or negative regard.

We generated ten items to measure students' perceptions of teachers' academic conditional regard. Five of them referred to teachers' academic conditional positive regard (e.g., I feel that when or if I do well in my exam, my teachers will accept me more) and the others focused on teachers' academic conditional negative regard (e.g., I feel that when or if I do not study well, my teachers will like me less). The method to obtain and to interpret variable scores were the same as one applied in measuring students' perception of parental academic conditional regard.

Five items from the contingencies of self-worth scale [7] were adjusted and used to assess students' academic contingent self-worth (e.g., I feel good about myself when or if I have a good academic achievement). Participants rated these items on a five-point Likert scale, ranging from 1 = strongly disagree to 5 = strongly agree. To obtain the variable score, we summed up these items. A higher total score reflected the stronger dependence of students' self-worth on academic achievement.

To dwell more into the influence of student's school alienation (SAL) on other factors including PCPR, PCNR, TCPR, TCNR AND CSW, we present the following regression:

SAL∼β0+β1*(PCPR)+β2*(PCNR)+β3*(TCPR)+β4*(TCPR)+β5*(TCNR)+β6*(CSW)+u

## Ethic Statements

Informed consent was obtained from all participants and their legal guardians before the two studies were proceeded. The work was conducted with the approval of EdLab Asia Educational Research and Development Centre’ Institutional Review Board. The IRB numbers for the study on perfectionism (Dataset A) and alienation (Dataset B) are No. 201009 and No. 200127, respectively.

## Declaration of Competing Interest

The authors declare that they have no known competing financial interests or personal relationships which have, or could be perceived to have, influenced the work reported in this article.
